# Downregulation of miR-522 suppresses proliferation and metastasis of non-small cell lung cancer cells by directly targeting DENN/MADD domain containing 2D

**DOI:** 10.1038/srep19346

**Published:** 2016-01-19

**Authors:** Tianze Zhang, Yingying Hu, Jin Ju, Liangyu Hou, Zhange Li, Dan Xiao, Yongchao Li, Jianyu Yao, Chao Wang, Yong Zhang, Linyou Zhang

**Affiliations:** 1 Department of Thoracic Surgery, The Second Affiliated Hospital of Harbin Medical University, Harbin 150086, China; 2 Department of Pharmacology (State-Province Key Laboratories of Biomedicine- Pharmaceutics of China, Key Laboratory of Cardiovascular Research, Ministry of Education), Harbin Medical University, Harbin 150081, China; 3 Department of Pharmacy, The First Affiliated Hospital of Harbin Medical University, Harbin 150001, China; 4 Department of Anesthesiology, The First Affiliated Hospital of Harbin Medical University, Harbin 150001, China

## Abstract

Non-small cell lung cancer (NSCLC), one of the most common causes of cancer-related death, is a worldwide public health problem. MicroRNAs (miRNAs) have recently been identified as a novel class of regulators of carcinogenesis and tumor progression, including miRNAs associated with NSCLC. This study aimed to explore the role of miR-522 in NSCLC and the mechanisms underlying this role. We report here that miR-522 expression was significantly increased in both human NSCLC tissues and cell lines. Furthermore, an MTT assay, 5-Ethynyl-2′-deoxyuridine (EdU) assay kit and flow cytometry confirmed that the inhibition of miR-522 suppressed NSCLC cells proliferation and induced apoptosis. Compared with miR-522 overexpression, miR-522 inhibitor markedly reduced cells migration and invasion, as indicated by wound-healing and transwell assays. In addition, a luciferase assay identified DENN/MADD domain containing 2D (DENND2D) as a direct target of miR-522. qRT-PCR and western blot analyses indicated the reciprocal expression of miR-522 and DENND2D in NSCLC tissue samples. DENND2D was involved in miR-522 induced proliferation and metastasis of NSCLC cells by a miRNA-masking antisense oligonucleotides (miR-mask) technology. These data highlight a novel molecular interaction between miR-522 and DENND2D, which indicates that targeting miR-522 may constitute a potential therapy for NSCLC.

## Introduction

Lung cancer is the leading cause of cancer mortality worldwide, and non-small cell lung cancer (NSCLC) accounts for approximately 80% of all lung cancer cases^1^. In 2013, approximately 270,000 individuals were predicted to die of lung cancer in the European Union^2^. Despite recent advances in both the diagnosis and treatment of NSCLC, the prognosis for lung cancer patients remains poor, and the 5-year survival rate for NSCLC patients remains at a low 15%^3^. Recent technical developments have focused on identifying specific gene expression signatures that are associated with tumor staging and patient prognosis to improve prognosis and therapy. However, the specific targets or genes remain unknown.

The DENN/MADD domain-containing (DENND) proteins regulate Rab GTPases and represent a newly recognized class of membrane trafficking proteins^4^. DENND proteins directly interact with Rab35 and function as guanine nucleotide exchange factors (GEFs) for this GTPase^5^,^6^. DENN/MADD domain containing 2D (DENND2D), a member of the DENND2 family, is located on chromosome 1p13.3 and encodes a 53-kDa protein that is a candidate tumor suppressor gene. Silencing via promoter hypermethylation regulates DENND2D in hepatocellular carcinoma (HCC)^7^, esophageal squamous cell carcinoma (ESCC)^8^ and gastric cancer (GC)^9^. DENND2D also reportedly suppresses the proliferation and tumorigenicity of NSCLC cells^10^. Nevertheless, the underlying mechanisms by which DENND2D is regulated require further exploration.

MicroRNAs (miRNAs) are short (19–25 nucleotides in length), non-coding, single-stranded RNAs that act as negative regulators of gene expression at the post-transcriptional level^11^. The study by Takamizawa *et al.*^12^ represents the first effort to relate miRNA expression to lung cancer. Since then, a growing number of studies have related miRNA expression to lung cancer, such as miRNAs in the let-7^12^ and miR-34 families^13^,^14^ or the cluster miR-17-92^15^. These miRNAs have been demonstrated to target genes that play important roles in lung carcinogenesis and have emerged as biomarkers for tumor diagnosis, prognosis and prediction of responses to treatment^16^.

miR-522 is a member of the chromosome 19 miRNA cluster (C19MC), a 100-kb, primate-restricted region that encodes 54 tandem miRNAs; this cluster is the largest miRNA cluster in the human genome^17^. miR-522 is reportedly upregulated in HCC and may contribute to tumor development^18^,^19^. Furthermore, miR-522 has been shown to enhance the ability of triple-negative breast cancer cells to survive detachment, invade through a membrane, and express mesenchymal genes, properties that are associated with metastasis^17^. The upregulation of miR-522 is associated with the development of glioblastoma and increased tumor cell proliferation *in vitro*^20^. However, the detailed role of miR-522 in NSCLC remains unknown. In this study, we first describe a potential role for miR-522 in the proliferation and metastasis of NSCLC. We also focus on the molecular mechanisms by which DENND2D, one of the direct targets of miR-522, may contribute to NSCLC development.

## Results

### miR-522 is highly expressed in NSCLC tissues and cell lines

**Figure 1:**
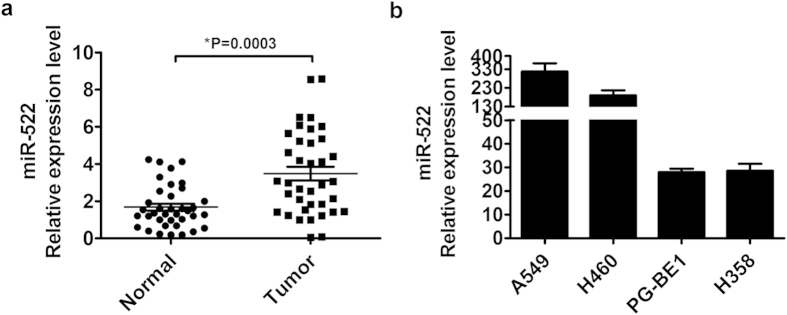
miR-522 expression in NSCLC tissues and cell lines by qRT-PCR. (**a**) The expression levels of miR-522 in 37 pairs of NSCLC tissues. *P < 0.05 vs Normal. (**b**) The relative quantities of miR-522 in 4 NSCLC cell lines were compared with the mean expression level of 10 normal lung specimens. The relative gene-expression level was calculated as follows: RQ = 2^−ΔΔCT^. Data are presented as the mean ± S.E.M.

To study the expression and significance of miR-522 in NSCLC carcinogenesis, we measured the expression of miR-522 in 37 pairs of NSCLC tissues and their matched normal lung tissues using qRT-PCR. miR-522 was significantly upregulated in NSCLC tissues compared with their matched normal tissues (Fig. 1a). In addition, miR-522 was dramatically upregulated in all 4 lung cancer cell lines, i.e., A549, H460, PG-BE1 and H358 (Fig. 1b). The expression of miR-522 was higher in A549 and H460 cells than in the other two cell lines. Thus, we focused on the A549 and H460 cell lines in subsequent experiments.

### miR-522 inhibitor suppresses cell proliferation and induces apoptosis in NSCLC cells

**Figure 2:**
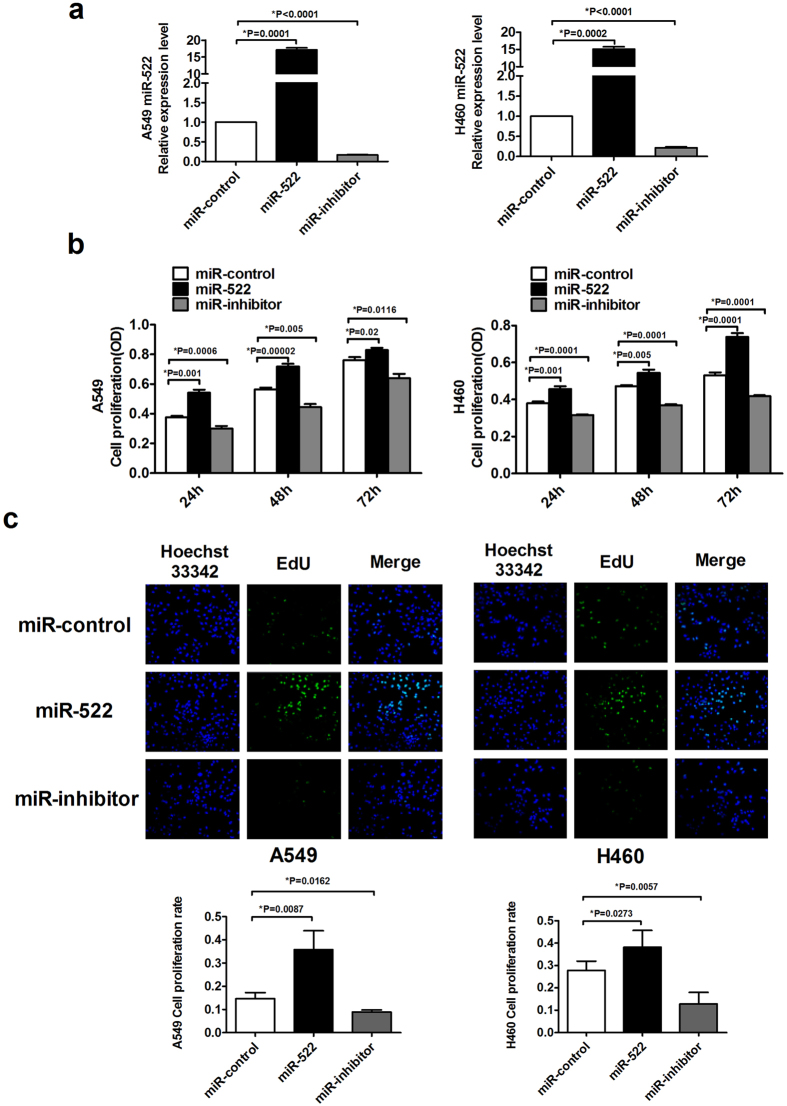
Effects of miR-522 on the proliferation of human NSCLC cells. (**a**) Cells were transfected with miR-522 or miR-522 inhibitor, and the expression of miR-522 was analyzed by qRT-PCR. *P < 0.05 vs miR-control. (**b**) The effects of miR-522 on NSCLC cells viability were measured with an MTT assay. A549 and H460 cells were transfected with miR-522 or miR-522 inhibitor for 24 h, 48 h, and 72 h. All independent experiments were performed 3 times. *P < 0.05 vs miR-control. (**c**) The proliferation of A549 and H460 cells was determined by EdU kit. Nuclei that double labeled with EdU (green) and Hoechst 33342 (blue) were considered to be new proliferative cells, assessed by fluorescence microscopy (200×). *P < 0.05 vs miR-control.

**Figure 3:**
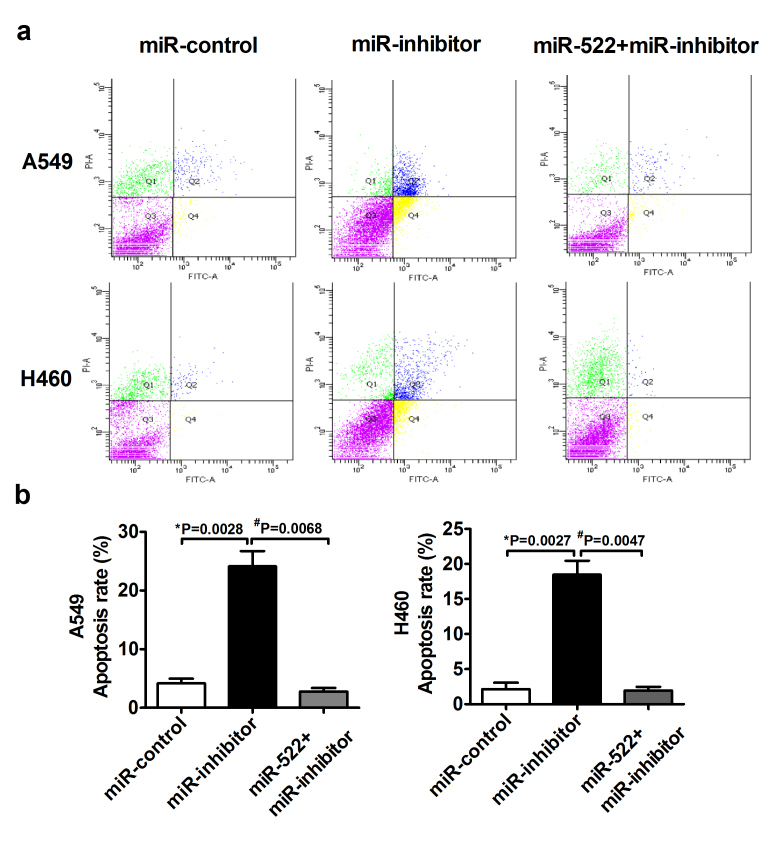
Effects of miR-522 on human NSCLC cells apoptosis. (**a**) Annexin V-FITC/propidium iodide staining and flow cytometry were performed to detect apoptosis. (**b**) The quantitative presentation of apoptotic cell populations. *P < 0.05 vs miR-control, ^#^*P* < 0.05 vs miR-inhibitor.

Based on the above results, we hypothesized a relationship between miR-522 and NSCLC cell proliferation. We first either overexpressed or inhibited miR-522 in A549 and H460 cells. The transfection efficiency was measured by qRT-PCR (Fig. 2a). As shown in Fig. 2b, the overexpression of miR-522 significantly increased the viability of A549 and H460 cells compared with their corresponding controls in different time points, whereas miR-522 inhibitor suppressed cell viability in both cell lines. To explore the functional role of miR-522 in NSCLC cell proliferation, we performed 5-Ethynyl-2′-deoxyuridine (EdU) assay kit. In Fig. 2c, more new proliferative cells double labeled with EdU and Hoechst 33342 were observed under transfection of miR-522 for 24 h compared with miR-control, whereas the number was markedly decreased after transfection of miR-522 inhibitor. The results for 48 h and 72 h were provided in Supplemental Fig. 2. To evaluate whether miR-522 inhibitor could induce apoptosis, we examined Annexin V-FITC/PI staining by flow cytometry. The transfection of miR-522 inhibitor dramatically increased the apoptosis rate, whereas co-transfection of miR-522 attenuated these beneficial effects (Fig. 3). However, transfection of miR-522 alone had no effect on apoptosis in Supplemental Fig. 3.

### miR-522 increases NSCLC cell migration and invasion

**Figure 4:**
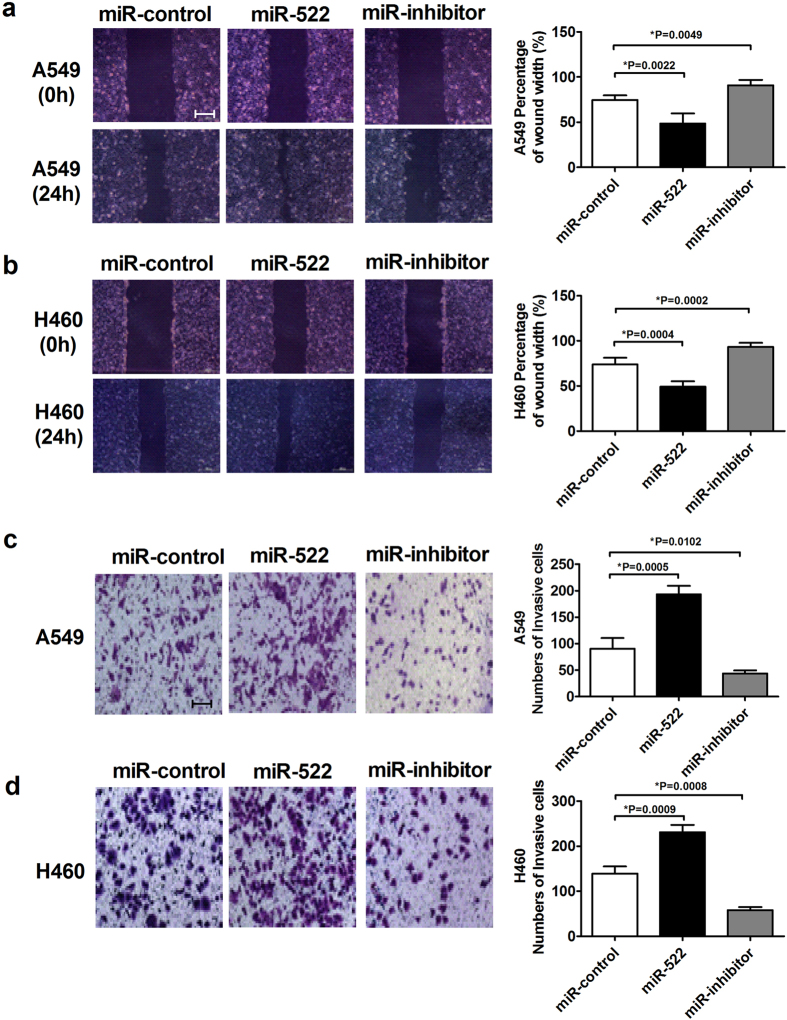
Effects of miR-522 on the migration of NSCLC cells. (**a**,**b**) Wound closure at 0 h and 24 h of A549 and H460 cells transfected with miR-522 or miR-522 inhibitor. Scale bar: 200 μm. (**c**,**d**) Transwell assays of A549 and H460 cells transfected with miR-522 or inhibitor. Magnification: 100×. Scale bar: 200 μm. *P < 0.05 vs miR-control.

**Figure 5:**
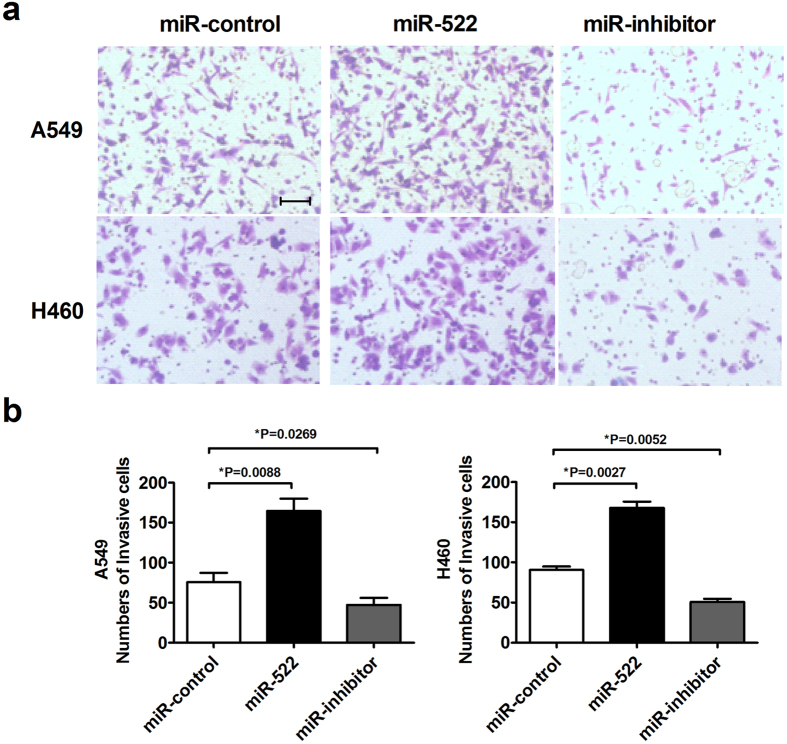
Effects of miR-522 on the invasion of NSCLC cells. (**a**) Transwell assays with Matrigel of A549 and H460 cells transfected with miR-522 or miR-522 inhibitor. (**b**) The quantitative presentation of the number of invasive cells. Scale bar: 200 μm. *P < 0.05 vs miR-control.

Cancer metastasis is the primary cause of cancer-associated death. The deregulation of cell migration during cancer progression determines the capacity of tumor cells to escape from the primary tumors and invade adjacent tissues to finally form metastases^21^. Therefore, we investigated the effects of miR-522 on the migration and invasion abilities of NSCLC cells. Figure 4a,b show that overexpression of miR-522 markedly promoted the mobility of A549 and H460 cells compared with the control group, whereas miR-522 inhibitor suppressed cell migration. The same results were obtained using transwell assays (Fig. 4c,d). Transwell assays with Matrigel were performed to evaluate the ability of cells to invade; exogenously increased miR-522 expression significantly increased the number of invasive cells, whereas miR-522 inhibitor had the opposite effect on NSCLC cells invasion (Fig. 5).

### miR-522 negatively regulates DENND2D expression

**Figure 6:**
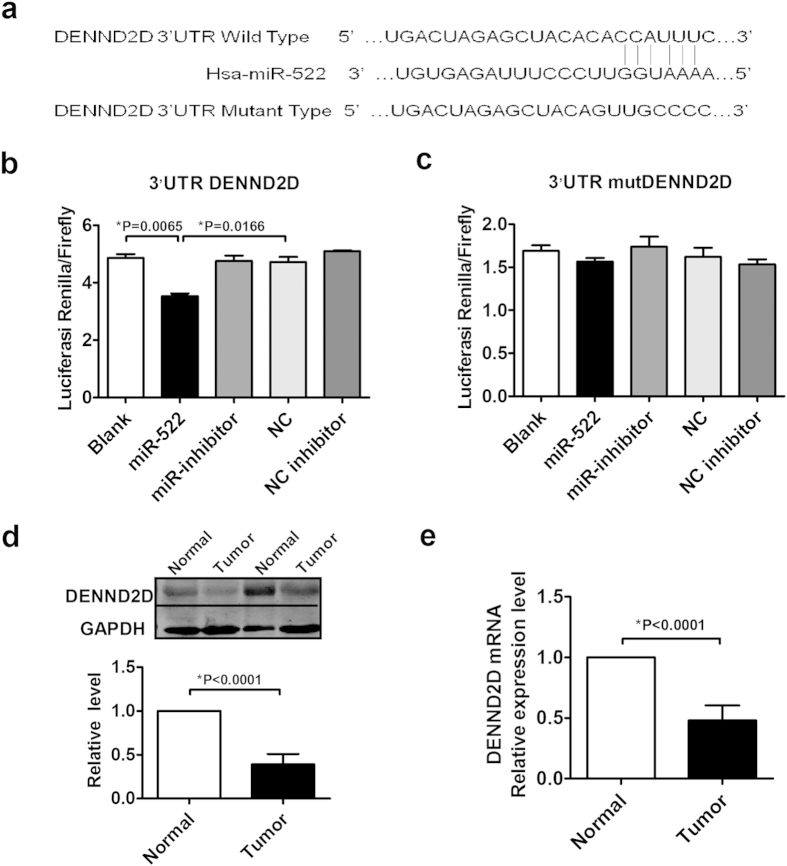
Identification of DENND2D as a miR-522 target gene. (**a**) The potential miR-522 binding sites in the DENND2D 3′UTR and the mutated sequences. (**b**,**c**) 293T cells were co-transfected with miR-522, miR-522 inhibitor, NC or NC inhibitor with WT or Mut 3′UTR. The luciferase activity was assayed 48 h after transient co-transfection. *P < 0.05 vs miR-522. (**d**) The comparison of protein levels of DENND2D in tumors and adjacent normal tissues. (**e**) The mRNA expression level of DENND2D in NSCLC tissues and their corresponding adjacent tissues. *P < 0.05 vs Normal.

To explore the molecular mechanisms by which miR-522 executes its function, we used several bioinformatic predictions, such as TargetScan and miRanda, to determine the potential target of miR-522. DENND2D, a tumor suppressor gene in several cancers, was identified as a direct and functional target of miR-522, as shown in Fig. 6a. We then used a relative luciferase reporter assay with the DENND2D 3′-untranslated region (3′UTR) to demonstrate that miR-522 dramatically inhibited the luciferase activity of the wild-type (WT) 3’UTR but not that of the mutant (Mut) 3’ UTR of DENND2D (Fig. 6b,c). Subsequently, we examined the DENND2D expression level in tumor tissues and their matched normal tissues and found that DENND2D was downregulated at the mRNA level, whereas miR-522 was upregulated in tumor tissues. Moreover, the protein level of DENND2D was decreased in NSCLC tissues compared with matched normal tissues (Fig. 6d,e).

### The expression of miR-522 negatively correlates with the DENND2D protein level in NSCLC cell lines

**Figure 7:**
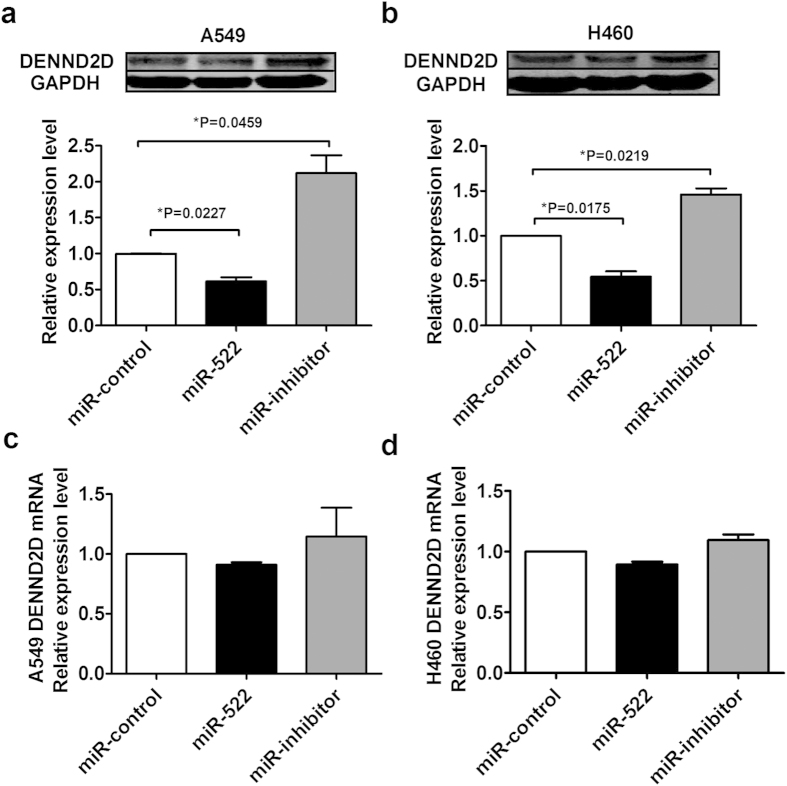
The correlation between miR-522 and DENND2D in NSCLC cells. (**a**) The protein level of DENND2D in A549 cells was analyzed by western blotting after transfection. (**b**) The protein level of DENND2D in H460 cells after transfection was analyzed by western blotting. (**c**) DENND2D mRNA expression level in A549 cells after transfection of miR-522 or miR-522 inhibitor, as measured by qRT-PCR. (**d**) The mRNA level of DENND2D in H460 cells measured by qRT-PCR after transfection. *P < 0.05 vs miR-control.

Based on the above results, we examined the expression of DENND2D responses to altered levels of miR-522 *in vitro*. The specific overexpression of miR-522 significantly downregulated the DENND2D protein level (Fig. 7a), whereas the suppression of miR-522 increased the DENND2D protein level (Fig. 7b). However, the mRNA level of DENND2D measured by qRT-PCR did not significantly change, indicating that miR-522 participates in the post-transcriptional regulation of DENND2D (Fig. 7c,d).

### DENND2D is involved in miR-522 induced NSCLC proliferation and metastasis

**Figure 8:**
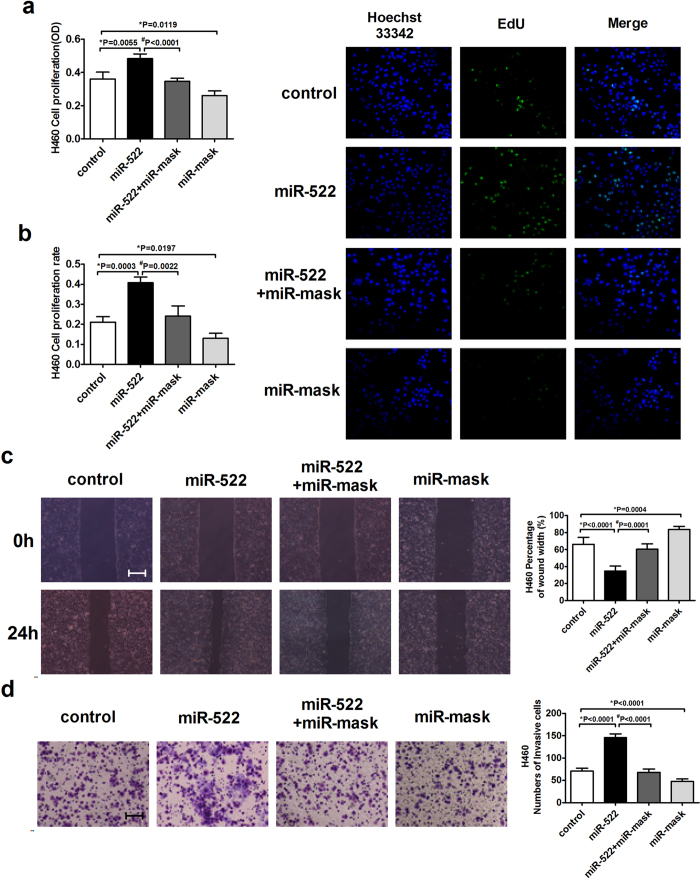
DENND2D is involved in miR-522 induced proliferation and metastasis of NSCLC cells. (**a**) The effects of miR-522 and miR-mask in H460 cell viability was measured with an MTT assay. (**b**) The proliferation of H460cells was determined by EdU kit, assessed by fluorescence microscopy (200×). (**c**) Wound closure at 0 h and 24 h in H460 cells infected with miR-522 or miR-mask. Scale bar: 200 μm. (**d**) Transwell assays with Matrigel in H460 cells infected with miR-522 or miR-mask. Magnification: 100×. Scale bar: 200 μm. *P < 0.05 vs miR-control, ^#^*P* < 0.05 vs miR-522.

To examine whether DENND2D is the key factor that miR-522 regulates proliferation and metastasis in NSCLC cells, we detected the effects of miR-522 with a corresponding miR-mask. In our study, the miR-mask was designed to be fully complementary to the target DENND2D sequence of miR-522. We confirmed that co-transfection of miR-522 and miR-mask acted against the overexpression of miR-522-induced NSCLC cell proliferation, and transfection of miR-mask alone suppressed the effect of endogenous miR-522 (Fig. 8a,b; Supplemental Fig. 4a & 4b). The overexpression of miR-522 effectively increased NSCLC cell migration and invasion, whereas miR-mask reversed the effects of miR-522 (Fig. 8c,d; Supplemental Fig. 4c & 4d).

## Discussion

In the present study, we reveal a novel role of miR-522 in the proliferation and metastasis of NSCLC cells. The expression of miR-522 was significantly upregulated in NSCLC tissues and cell lines. The inhibition of miR-522 effectively decreased proliferation and induced apoptosis in NSCLC cells. Furthermore, the overexpression of miR-522 dramatically increased the ability of NSCLC cells to migrate and invade. The mechanism by which miR-522 affects NSCLC cells was associated with changes in the expression of DENND2D. miR-522 may function as an oncogene by directly targeting DENND2D to regulate NSCLC.

miRNAs, which induce mRNA degradation or inhibit translation via imperfect hybridization with the 3′-UTRs of target mRNAs, have been reported to play vital roles in tumor progression^22^. Dysregulated miRNA expression affects nearly all aspects of cancer progressions, including cell proliferation, apoptosis, migration and invasion, and miRNAs can function as either tumor suppressors or oncogenes^23^,^24^. In NSCLC, several miRNAs, such as members of the let-7 family, miR-126, miR-145, or miR-34, have been identified as tumor suppressors and potential prognostic markers^12^,^13^,^14^,^25^,^26^. In addition, miR-17-92, miR-21, and miR-31 were found to be NSCLC oncogenes^15^,^27^,^28^. miR-522, an oncogene, has been documented to promote tumor activity *in vitro* and/or *in vivo* in HCC, breast cancer and glioblastoma^17^,^18^,^19^,^20^. However, the detailed role of miR-522 in NSCLC remains unknown. To better understand the role of miR-522 in NSCLC, we first analyzed the effect of miR-522 expression on the tissues of NSCLC patients and four NSCLC cell lines. miR-522 was significantly upregulated, indicating that miR-522 may play an important role in NSCLC carcinogenesis and progression.

Because miR-522 expression was upregulated in NSCLC tissues and cells, we identified the functional roles of miR-522 in all aspects of NSCLC progression, including cell proliferation, apoptosis, migration and invasion. Consistent with a previous study reported by Zhang *et al.*^20^, miR-522 promoted A549 and H460 cell proliferation, an effect that could be reversed by miR-522 inhibitor. Furthermore, a previous study showed that miR-522 induces G1 cell-cycle arrest and causes cells to detach without anoikis, become invasive, and express mesenchymal genes^17^. These findings are particularly interesting because our data show that the inhibition of miR-522 effectively induced apoptosis in A549 and H460 cells. Overexpression of miR-522 dramatically increased the migration and invasiveness of NSCLC cells, whereas miR-522 inhibition reversed these effects.

To clarify the underlying molecular mechanisms by which miR-522 participates in NSCLC progression, we used ten different types of prediction software to predict gene targets for miR-522, which identified DENND2D as a potential downstream target. DENND2D, a regulator of Rab GTPases, is a member of the DENND2 family^5^. The downregulation of DENND2D has been observed not only in NSCLC cell lines and lung squamous cell carcinoma (SCC) tissues but also in immortalized human bronchial epithelial (IHBE) cell lines and precancerous lesions, indicating that the downregulation of DENND2D may be an early event in lung cancer^10^. The overexpression of DENND2D significantly suppressed the proliferation of NSCLC cells *in vitro* and *in vivo* by inducing apoptosis^10^. Moreover, DENND2D is a candidate tumor suppressor gene that is regulated by silencing via promoter hypermethylation; DENND2D also serves as a novel biomarker for the early recurrence of HCC, ESCC and GC. In present study, we confirmed that the expression of DENND2D was reduced in NSCLC tissues compared with their matched normal tissues. A luciferase assay showed that miR-522 directly bound to the 3′-UTR of DENND2D. The overexpression of miR-522 in A549 and H460 cells was sufficient to suppress the expression of DENND2D. However, ectopic miR-522 expression decreased DENND2D only at the protein level and not at the mRNA level, indicating that it did not degrade but, rather, inhibited DENND2D mRNA translation. To certify that DENND2D is required for miR-522 to mediate its functions, we used a miR-mask technology. A miR-mask does not directly interact with its target miRNA but binds to the binding site of that miRNA in the 3′UTR of the target mRNA by fully complementary mechanism to appropriately study the specific outcome of regulation of the target gene by the miRNA, instead of binding to the target miRNA like the miRNA inhibitor^29^. From result showed in Fig. 8, we demonstrated that miR-mask designed to be fully complementary to the target DENND2D sequence of miR-522 reversed the effects of miR-522 on NSCLC cell proliferation and metastasis, indicating that miR-522 may function as an oncogene in NSCLC cells by directly targeting DENND2D.

The aim of the present study was to evaluate the roles of miR-522 on proliferation and metastasis/invasion in non-small cell lung cancer cell lines and elucidate the mechanisms underlying the effects at the cellular and molecular levels. We believe that our approaches have allowed us to generate sufficient data in support of our conclusions. Moreover, the fact that miR-522 was also found upregulated in human NSCLC tissues as in the cell lines is suggestive of the potential role of this miRNA in the tumorigenesis. However, it should be pointed out that our *in vitro* observations may not be readily applied to *in vivo* situations in the absence of *in vivo* studies and the possible role of miR-522 in pathogenesis of lung cancer merits further studies using animal models of tumors. Nonetheless, like numerous published studies with similar approaches at the cellular level, our findings serve to provide clues for the miR-522 functioning in tumor growth and metastasis of NSCLC.

Overall, our study is the first to show that miR-522 plays an important role in NSCLC carcinogenesis by affecting cell proliferation, apoptosis, migration and invasion. As a consequence, the pathological loss of miR-522 may suppress tumorigenesis by directly regulating the tumor suppressor gene DENND2D. miR-522 may therefore represent a novel therapeutically relevant cellular target for the treatment of NSCLC patients.

## Methods

### Tumor and normal tissue samples

For the human tissue samples, the methods were carried out in accordance with the approved guidelines by the Ethics and Scientific Committees of Harbin Medical University. A total of 37 fresh samples and corresponding normal samples were obtained from patients at the Second Affiliated Hospital of Harbin Medical University from July 2013 to January 2015. Overall, ten normal lung specimens were obtained from patients who underwent surgery for benign lung disease. Informed consent was obtained from all patients prior to tissue collection. No patients had received radiation therapy or chemotherapy prior to surgery. The clinicopathological information of patients, including age, gender, histological type, stage and lymph node metastasis, was obtained from patient records and is summarized in Table 1.

### Cell culture and transfection

Four cell lines (A549, H460, PG-BE1, and H358) were purchased from ATCC. The cells were grown in Dulbecco’s modified eagle’s medium (DMEM) containing 10% fetal bovine serum (FBS) at 37 °C in 5% CO_2_. The cells were starved in serum-free medium for 24 h, and then transiently transfected with miR-522 mimics, miR-522 inhibitors or negative controls, and miR-mask (RiboBio Co., Ltd., Guangzhou, Guangdong, China) using X-treme GENE siRNA transfection reagent (catalog #04476093001; Roche, Indianapolis, USA) according to the manufacturer’s instructions.

### Quantitative real-time PCR (qRT-PCR)

Total RNA was harvested from tissues and cells using TRIzol reagent (Invitrogen, CA, USA) according to the manufacturer’s protocols. cDNA synthesis was performed using a High Capacity cDNA Reverse Transcription Kit (Applied Biosystems, Carlsbad, CA, USA; Cat. no. 4368814) according to the manufacturer’s instructions. The levels of miR-522 and DENND2D mRNA were determined using a SYBR Green I incorporation method and an ABI 7500 fast Real Time PCR system (Applied Biosystems, USA). U6 and GAPDH were used as internal controls for miR-522 and DENND2D, respectively^30^. PCR primer for miR-522 (5′-AAAAUGGUUCCCUUUAGAGUGU-3′) was designed by RiboBio Co., Ltd. (Guangzhou, China). The following primers were used: DENND2D forward primer, 5′-ATCTTTGCCTCTGCCGTGCT-3′; DENND2D reverse primer, 5′-GGACAACAGGGATGTAGGTG-3′; GAPDH forward primer, 5′-AAGAAGGTGGTGAAGCAGGC-3′; GAPDH reverse primer, 5′-TCCACCACCCAGTTGCTGTA-3′; U6 forward primer, 5′-GCTTCGGCAGCACATATACTAAAAT-3′; U6 reverse primer, 5′-CGCTTCACGAATTTGCGTGTCAT-3′.

### MTT assay

A 3-(4,5-dimethylthiazol-2-yl)-2,5-diphenyltetrazolium bromide (MTT) assay was used to measure NSCLC cell viability, as described previously^31^. Briefly, cells (2 × 10^4^ cells/well) were seeded in each well of a 96-well plate in 100 μl of culture medium, and the cells were allowed to grow for 24 h, 48 h, and 72 h. Twenty microliters of MTT solution was added to each well, and the cells were then incubated for 4 h. The medium was then discarded, followed by the addition of 150 μl of DMSO. Finally, the absorbance at 490 nm was recorded.

### EdU assay kit

Cells plated on coverslips in 24-well culture plates were treated as experiment design. The proliferation of A549 and H460 cells were detected by EdU kit (RiboBio, China) according to the manufacturer’s instructions^32^. A fluorescence microscope (Olympus, Japan) was used to acquire the images. Nuclei that double labeled with EdU and Hoechst 33342 were considered to be positive cells.

### Annexin V-FITC Apoptosis Detection kit

An Annexin V-FITC Apoptosis Detection kit was used according to the manufacturer’s instructions to detect apoptosis following treatment with miR-522 or miR-522 inhibitor (Beyotime, Shanghai, China). Briefly, the cells were digested with 0.25% trypsin and collected by centrifugation after each type of treatment. After being washed twice with PBS, the cells were stained with Annexin V-FITC for 15 min and propidium iodide (PI) for 5 min. Apoptotic cells were identified by flow cytometry. Q4, Annexin V-FITC^+^/PI^−^, early apoptosis; Q2, Annexin V-FITC^+^/PI^+^, late apoptosis^33^.

### Migration and invasion assays

To analyze wound healing, the cells were seeded in six-well plates with DMEM medium. After 48 h, the cell monolayer was wounded using a plastic pipette tip. The cells were then rinsed with PBS and cultured with serum-free DMEM for 24 h. The wound closure was observed and photographed under a microscope. For the Transwell assays, 8-μm pore size chambers (Corning, NY, USA), were used with or without an insert coated with Matrigel (BD Bioscience). Twenty-four hours after transfection, 1 × 10^5^ cells in serum-free medium were added to the upper chamber. The lower chamber was filled with 10% FBS DMEM. After 18 h of incubation, the cells remaining on the upper surface of the membrane were removed, whereas the cells that had invaded through the membrane were fixed with 0.1% paraformaldehyde, stained with 0.1% crystal violet, imaged, and counted under a microscope (Olympus, Japan)^34^.

### Western blot analysis

Protein samples were extracted with RIPA buffer supplemented with protease inhibitors and quantified using the BCA method (Beyotime, Shanghai, China). For the western blot analysis, 100-μg protein samples were fractionated by SDS-PAGE (10% SDS-polyacrylamide gel) and transferred to PVDF membranes. Primary antibodies against total DENND2D (Abcam, Cambridge, MA, USA) were used, and GAPDH (anti-GAPDH from Kangcheng Inc., Shanghai, China) was used as an internal control. The blotted proteins were detected and quantified using an Odyssey Infrared Imaging System (LI-COR, Lincoln, USA.)^32^,^35^.

### Luciferase reporter assay

Luciferase reporter assays were performed as previously described^36^,^37^. Briefly, a luciferase reporter containing the WT or Mut 3′-UTR of DENND2D was constructed using psi-CHECK2 vectors (Promega, Madison, MI, USA). 293T cells (2 × 10^4^ cells/well) were cultivated in a 24-well plate and co-transfected with miR-522 mimics or miR-522 inhibitors and plasmid using Lipofectamine 2000 reagent. Forty-eight hours after transfection, the luciferase activity was measured with a Dual-Luciferase Reporter Assay System.

### Statistical analysis

Group data are expressed as the mean ± S.E.M. Differences between two groups were assessed using Student’s t-test. Multiple groups were compared with a one-way ANOVA accompanied by Bonferroni’s Multiple Comparison Test. *P* < 0.05 was considered to indicate a significant difference. Data were analyzed using the GraphPad Prism 5.0 software.

## Additional Information

**How to cite this article**: Zhang, T. *et al.* Downregulation of miR-522 suppresses proliferation and metastasis of non-small cell lung cancer cells by directly targeting DENN/MADD domain containing 2D. *Sci. Rep.* **6**, 19346; doi: 10.1038/srep19346 (2016).

## Supplementary Material

Supplementary Information

## Figures and Tables

**Table 1:** Relationship between miR-522 expression and clinicopathological parameters in 37 fresh samples of lung cancers.

miR-522			
Variable	Patiens	High expression	Low expression
Age(years)			
≥60	19	8(42.1%)	11(57.9%)
<60	18	8(44.4%)	10(55.6%)
Gender			
Male	26	11(42.3%)	15(57.7%)
Female	11	5(45.4%)	6(54.6%)
Smoking history			
Smoker	25	14(56%)	11(44%)
None smoker	12	5(41.7%)	7(58.3%)
Histology			
SCC	16	5(31.2%)	11(68.8%)
AC	21	11(52.4%)	10(47.6%)
Stage			
I	11	4(36.3%)	7(63.7%)
II	19	11(57.9%)	8(42.1%)
III	7	3(42.8%)	4(51.2%)
Lymph node status			
No Metastasis	15	7(46.7%)	8(53.3%)
Metastasis	22	12(54.5%)	10(45.5%)
