# Supplementary material for: Downregulation of miR-522 suppresses proliferation and metastasis of non-small cell lung cancer cells by directly targeting DENN/MADD domain containing 2D
Source: Sci Rep. 2016 Jan 19;6:19346. doi: 10.1038/srep19346 (PMC4726064; doi:10.1038/srep19346)
Supplement: Supplementary Information [file srep19346-s1.pdf]

Supplementary materials

Downregulation of miR-522 suppresses proliferation and metastasis of non-small cell lung cancer cells by directly targeting DENN/MADD domain containing 2D

Tianze Zhang[1], Yingying Hu[2, 3], Jin Ju[2], Liangyu Hou[2], Zhange Li[2], Dan Xiao[2], Yongchao Li[1], Jianyu Yao [1], Chao Wang[4], Yong Zhang[*, 2], Linyou Zhang[*, 1]

[1]Department of Thoracic Surgery, The Second Affiliated Hospital of Harbin Medical University, Harbin 150086, China

[2]Department of Pharmacology (State-Province Key Laboratories of Biomedicine-Pharmaceutics of China, Key Laboratory of Cardiovascular Research, Ministry of Education), Harbin Medical University, Harbin 150081, China

[3]Department of Pharmacy, The First Affiliated Hospital of Harbin Medical University, Harbin 150001, China

[4]Department of Anesthesiology, The First Affiliated Hospital of Harbin Medical University, Harbin 150001, China.

*Correspondence to: Linyou Zhang, Department of Thoracic Surgery, the Second Affiliated Hospital of Harbin Medical University, China. Tel: 86-451-86605499, Fax: 86-451-86605499, E-mail: lyzhang@hotmail.com; or Yong Zhang, Department of Pharmacology (State-Province Key Laboratories of Biomedicine-Pharmaceutics of China, Key Laboratory of Cardiovascular Research, Ministry of Education), Harbin Medical University, Harbin 150081, China. Tel: 86-451-86671354, Fax: 86-451-86671354, E-mail: hmuzhangyong@hotmail.com.

Supplemental Figures

Supplemental Figure 1

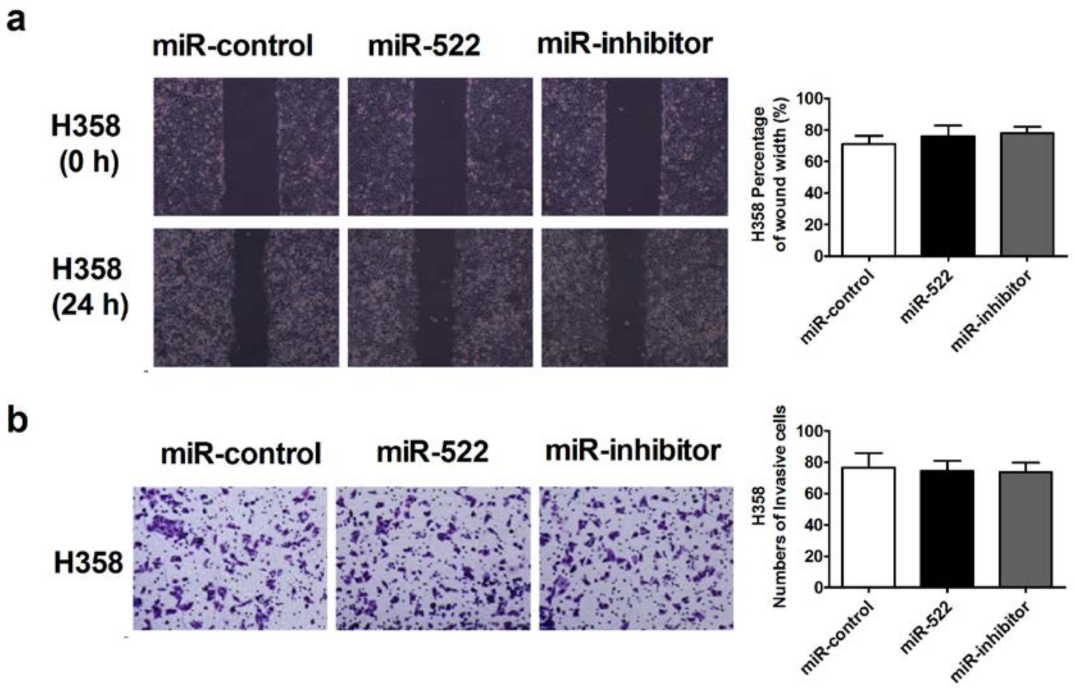

Supplementary Figure 1. Effects of miR-522 on the migration and invasion of H358 cells. (a) Wound closure at 0 h and 24 h of H358 cells that were infected with miR-522 or miR-inhibitor. The quantitative presentation of percentage of wound width. (b) Transwell assays with Matrigel of H358 cells infected with miR-522 or miR-inhibitor. Magnification: 100×. The quantitative presentation of the number of invasive cells. *$P < 0.05$ vs miR-control.

Supplemental Figure 2

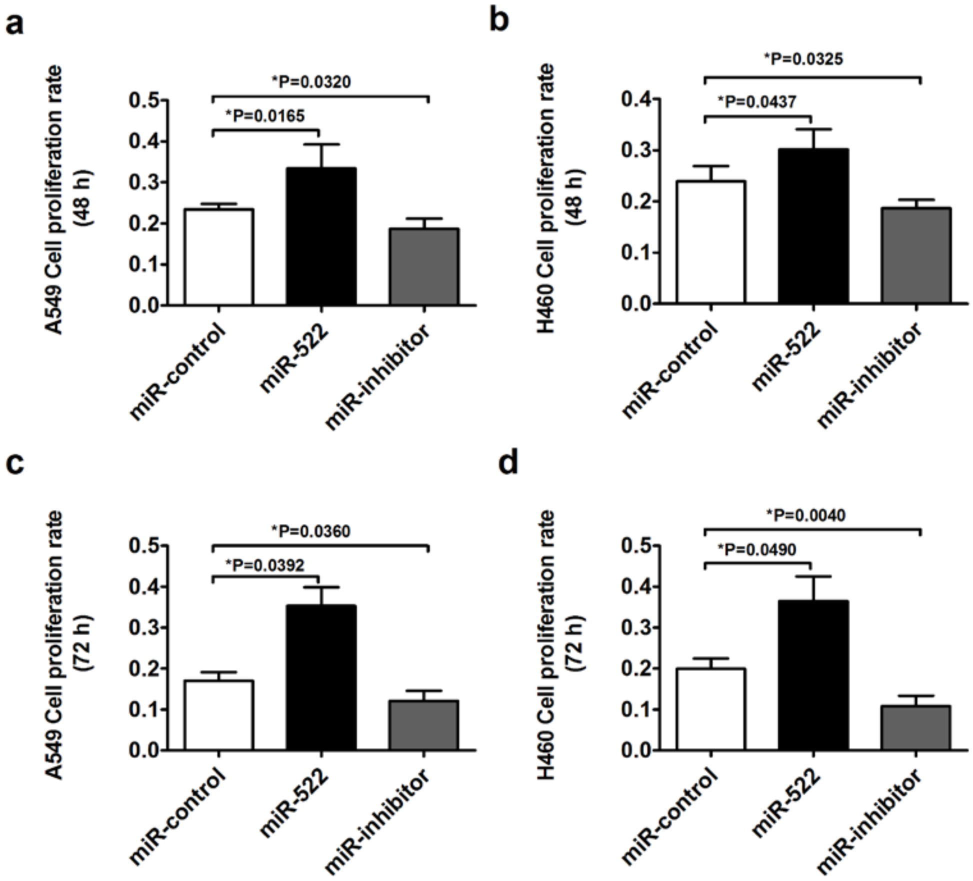

Supplementary Figure 2. Effects of miR-522 on the proliferation of A549 and H460 cells. (a-d) The proliferation of A549 and H460 cells infected with miR-522 or miR-inhibitor for 48 h and 72 h was determined by EdU kit. Nuclei that double labeled with EdU (green) and Hoechst 33342 (blue) were considered to be new proliferative cells, assessed by fluorescence microscopy (200×). The quantitative presentation of cell proliferation rate. *P < 0.05 vs miR-control.

Supplemental Figure 3

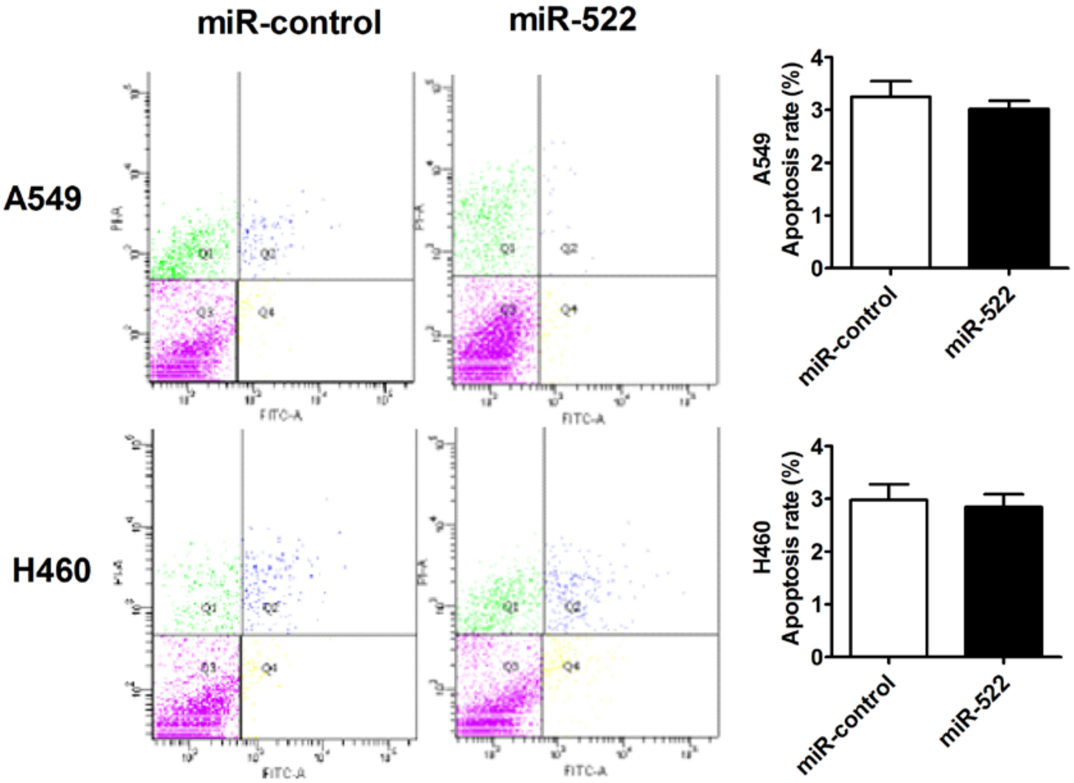

Supplementary Figure 3. Effects of miR-522 on human NSCLC cells apoptosis. Annexin V-FITC/propidium iodide staining and flow cytometry were performed to detect the effects of miR-522 on apoptosis. The quantitative presentation of apoptotic cell populations.

Supplemental Figure 4

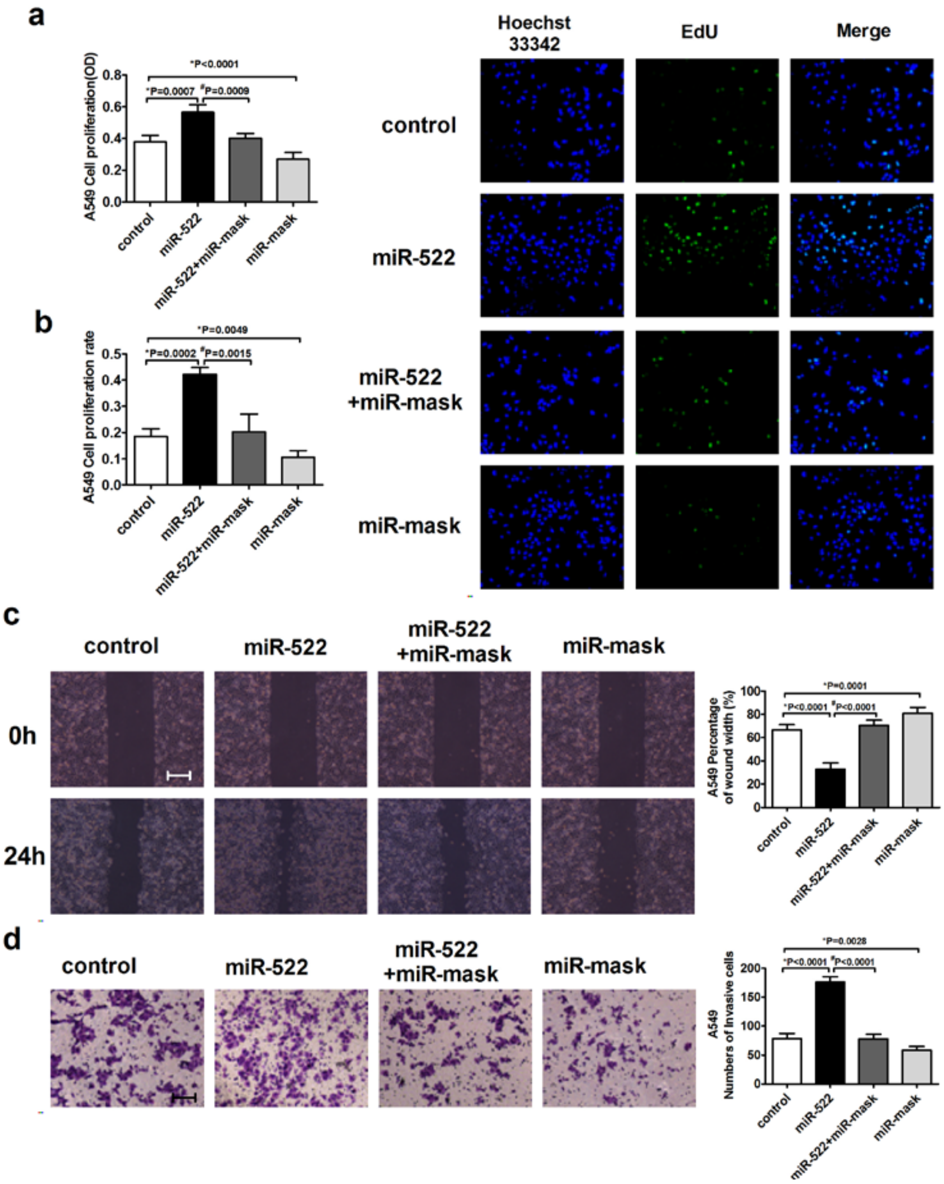

Supplementary Figure 4. DENND2D is involved in miR-522 induced A549 cells proliferation and metastasis. (a) The effects of miR-522 and miR-mask in A549 cell viability was measured with an MTT assay. (b) The proliferation of A549 cells was determined by EdU kit, assessed by fluorescence microscopy (200×). (c) Wound closure at 0 h and 24 h in A549 cells infected with miR-522 or miR-mask. Scale bar: 200 μm. (d) Transwell assays with Matrigel in A549 cells infected with miR-522 or miR-mask. Magnification: 100×. Scale bar: 200 μm. **P* < 0.05 vs miR-control, #*P* < 0.05 vs miR-522

Supplemental Figure 5

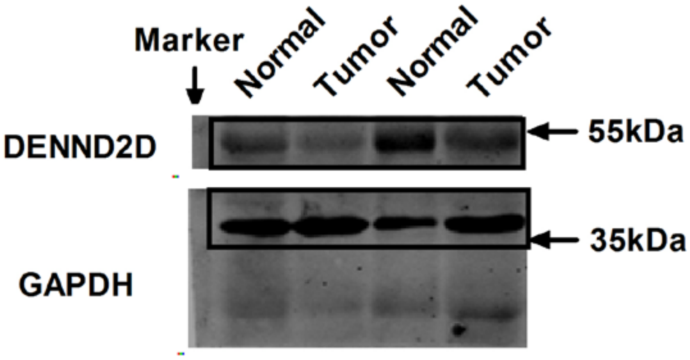

Supplemental Figure 5. Full-length blots of Figure 6(d) in the main text. The protein levels of DENND2D in tumors and adjacent normal tissues detected by western blot.

Supplemental Figure 6

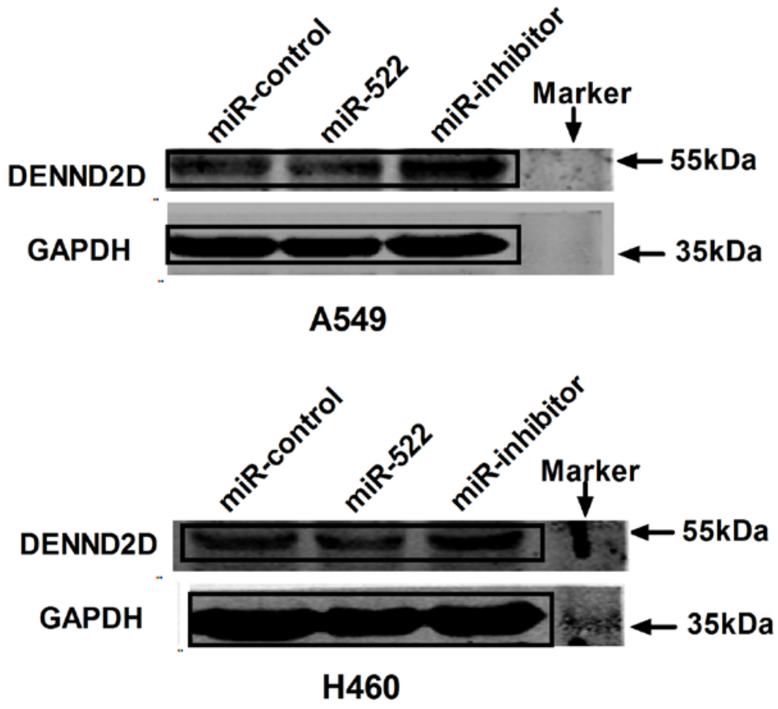

Supplemental Figure 6. Full-length blots of Figure 7(a) and 7(d) in the main text.

The protein level of DENND2D in A549 and H460 cells detected by western blot.
